# Supplementary material for: Exploring factors contributing to adolescent suicide using psychological autopsy - A scoping review
Source: PLoS One. 2026 May 27;21(5):e0348014. doi: 10.1371/journal.pone.0348014 (PMC13215618; doi:10.1371/journal.pone.0348014)
Supplement: Attachment — Submitted filename: Response to reviewers.doc [file pone.0348014.s003.doc]

**Reply to the reviewers’ comments**

| Reviewer Number | Original comments of the reviewer | Reply by the author(s) | Changes done on page number and line number |
| --- | --- | --- | --- |
| 1 | but the review's transparency and replicability would be strengthened with greater clarity regarding the search strategy, inclusion criteria, and geographic scope | Added the details regarding the search strategy, inclusion criteria and geographical scope | Search strategy in page no 7, lines from 129-133. Inclusion and exclusion criteria in table 1. Page 6, line 120 |
| 1 | The article could do a better job of explaining how thematic synthesis was carried out and whether the calibre of the included studies was evaluated, even though the four emergent themes are clearly categorized | Added details about the narrative synthesis | Page no 8, line 164-169  Page no 8, line 154-158 |
| 1 | Furthermore, there is a disconnect between the objectives and the results that are presented because, although the suggested conceptual framework is mentioned as an objective, it is not explained in the article. | Added a description about the conceptual framework | Page no 20, line 358-370 |
| 1 | Although the conclusion is sound, more precise implications for practice, policy, and future research would be beneficial | Added a section on Implication for Policy, practice and future research | Page no 22, line 427-434 |
| 1 | It is informative and cohesive overall, but more methodological detail would improve it. | Improvements are made to the methodology section | Page no 5 page no 8 |
| Journal requirements1. | Please ensure that your manuscripts meet PLOS ONE style requirements including those of file naming. | Modified. |  |
| 2 | PLOS requires an ORCID Id for the corresponding author in editorial manager on papers submitted. | Added the corresponding author and ORCID id |  |
| 3 | About data availability statement. | There is no raw data in this, only published data used in this review. |  |
| 4&5 | About restrictions to data sharing for this study. | Only published data used in this study |  |
| 6&7 | About reference list | No retracted paper has been cited in this study |  |
| Editor comments 1 | Original Title | Modified to “Exploring Factors Contributing to Adolescent Suicide Using Psychological Autopsy: A Scoping Review" | Page no.1, line 1 highlighted |
|  | Abstract | Modified to "...necessitates the urgent development of targeted prevention strategies." | Page no 2, line 28 highlighted |
|  | Objective | Revised to "...synthesis of the trajectories leading to adolescent suicide." | Page no 2, line 33,34 highlighted |
| 2 | Introduction | Revised to "Each completed suicide is estimated to prompt at least 25 additional suicide attempts..."  Revised to "During this phase, adolescents develop self-awareness and identity, while facing increasing obligations..." | Page no 3, line 56 highlighted  Page no 3, line 68&69 highlighted |
| 3 | Methodology | Modified to "Studies confirming a clear verdict of suicide."  Modified to "Any disagreements arising between reviewers..." | Page no 6, line 120 highlighted  Page no 7, line 146, 147 highlighted |
| 4 | Results (Individual factors) | Revised to "...one of the most frequent and significant individual risk factors..."  Revised to "...encompassing alcohol and other unspecified substances..." | Page no 13, line 212 Highlighted  Page no 14, line 227, 228 highlighted |
|  |  | Modified to "...another focusing on social network addiction..." | Page no 14, line 235 highlighted |
